# Lateral Costal Artery: Clinical Importance of an Accessory Thoracic Artery

**DOI:** 10.21470/1678-9741-2017-0252

**Published:** 2018

**Authors:** Ünsal Vural, Ahmet Arif Aglar, Sinan Sahin, Mehmet Kizilay

**Affiliations:** 1 Department of Cardiovascular Surgery, Dr. Siyami Ersek Thoracic and Cardiovascular Surgery Training and Research Hospital, Istanbul, Turkey.

**Keywords:** Mammary Arteries, Subclavian Steal Syndrome, Thoracic Wall/blood supply, Internal Mammary-Coronary Artery Anastomosis

## Abstract

The lateral costal artery has sometimes been identified as the culprit for the "steal phenomenon" after coronary artery bypass grafting, besides being occasionally used for myocardial revascularization. Its branches make anastomoses with the internal thoracic artery through lateral intercostal arteries. We aim to report, on three cases, the clinical significance of a well-developed lateral costal artery after coronary artery bypass grafting. Two out of three patients who underwent coronary artery bypass graft surgery in our center between June 2010 and August 2017, applied to us with stable angina pectoris, while the third one was diagnosed with acute coronary syndrome after applying to the emergency department. In coronary cineangiography, in all three cases, a well-developed accessory vessel arising from the proximal 2.5 cm segment of the left internal thoracic artery coursed as far as the 6^th^ rib was detected, and it was confirmed to be the lateral costal artery. A stable angina pectoris in two of the patients was thought to be the result of steal phenomenon caused by the well-developed lateral costal artery. In the two cases with stable angina pectoris the lateral costal artery was obliterated via coil embolization. In the other case with the proximal left anterior descending artery stenosis, before percutaneous coronary intervention, the lateral costal artery was obliterated via coil embolization and the occluded subclavian artery was stented. Routine visualization in cineangiography and satisfactory surgical exploration of the left internal thoracic artery could be very helpful to identify any possible accessory branch of the left internal thoracic artery like the lateral costal artery.

**Table:** 

Abbreviations, acronyms & symbols		
**CABG**		**= Coronary artery bypass grafting**
**ECG**		**= Electrocardiography**
**LAD**		**= Left anterior descending artery**
**LCA**		**= Lateral costal artery**
**LITA**		**= Lateral internal thoracic artery**
**SCA**		**= Subclavian artery**

## INTRODUCTION

The first description of the lateral costal artery (LCA) was in 1730 by Heister, who called it the lateral internal thoracic artery (LITA)^[1]^. The famous anatomist Henle described it further as "arising from the internal thoracic near its entrance into the thorax and descending on the inner surface of four to six upper ribs and anastomosing with the corresponding intercostal arteries". In the same study, its risky location in terms of thoracentesis and various surgical procedures was also underlined^[1]^. Lateral costal artery (LCA) rises as a first branch of the LITA in 92% of the population. It is present 5.5% bilaterally and 11.1% unilaterally^[2]^. The mean distance between internal thoracic artery origin and lateral costal branch origin is 2.3 cm and 2.9 cm on the right and left side of the anterior thoracic wall, respectively. Mean diameter of the LCA is found to be 1.74±0.8 mm^[2]^. It has sometimes been identified as culprit for the "steal phenomenon" after coronary artery bypass grafting (CABG) and the artery itself is occasionally used for myocardial revascularization^[3]^. Embryologically, this artery, like the normal parietal arteries of the trunk, might form a longitudinal channel connecting the intersegmental arteries^[3]^.

In spite of advanced surgical techniques, it's not possible to improve LITA exploration to divide all its side branches. Ligation of the 1^st^ intercostal and more proximal branches of the LITA, which have superiority in left ventricular revascularization with 1A level of evidence, is of great importance to prevent "steal phenomenon". It's reported that non-ligated side branch frequency in coronary angiographies performed in patients who underwent coronary artery bypass grafting (CABG) is between 9-25%^[4]^.

## CASES

**Fig. 1:**
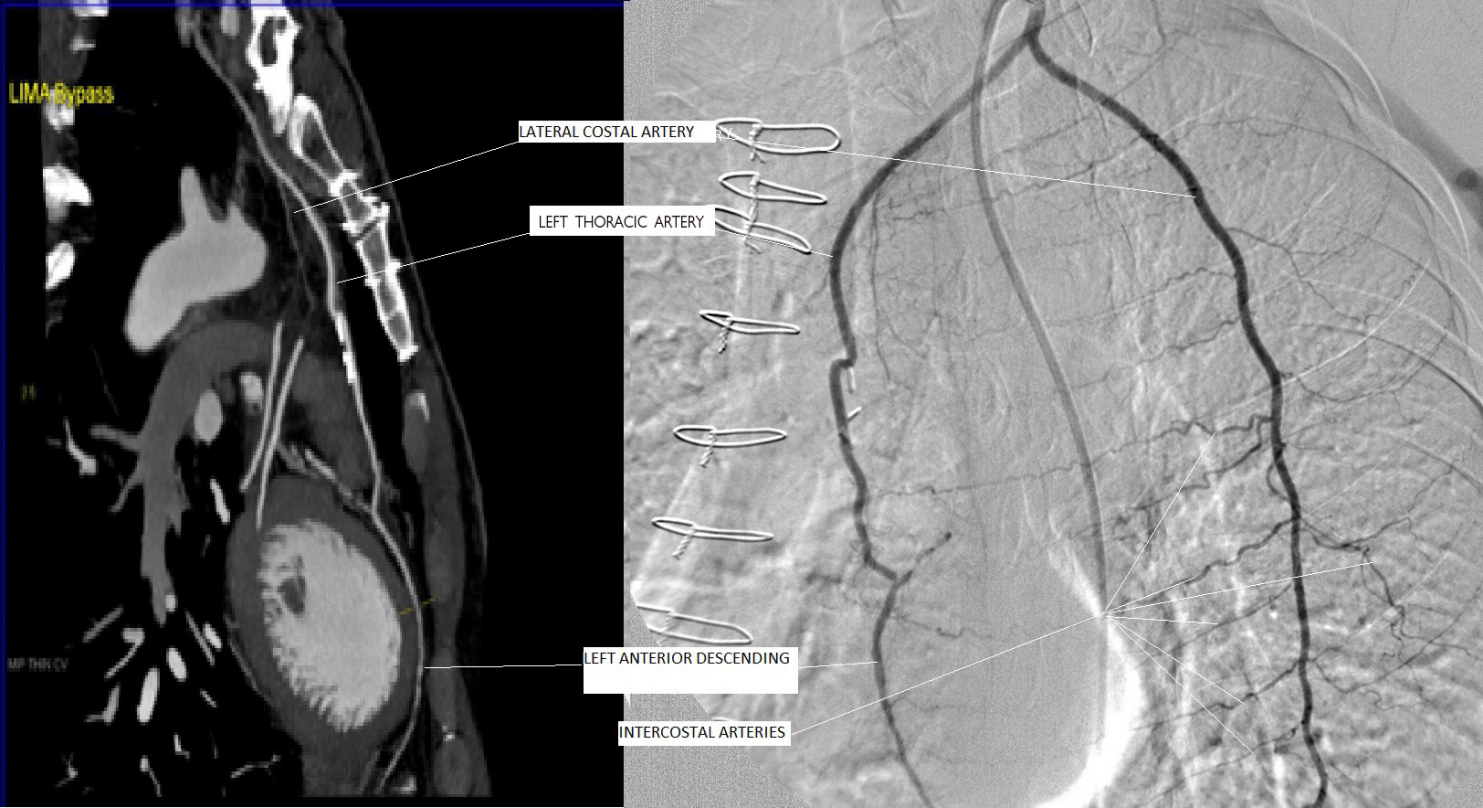
Computed tomography e cineangiographic view of the undivided LCA branch of the LITA.

**Fig. 2:**
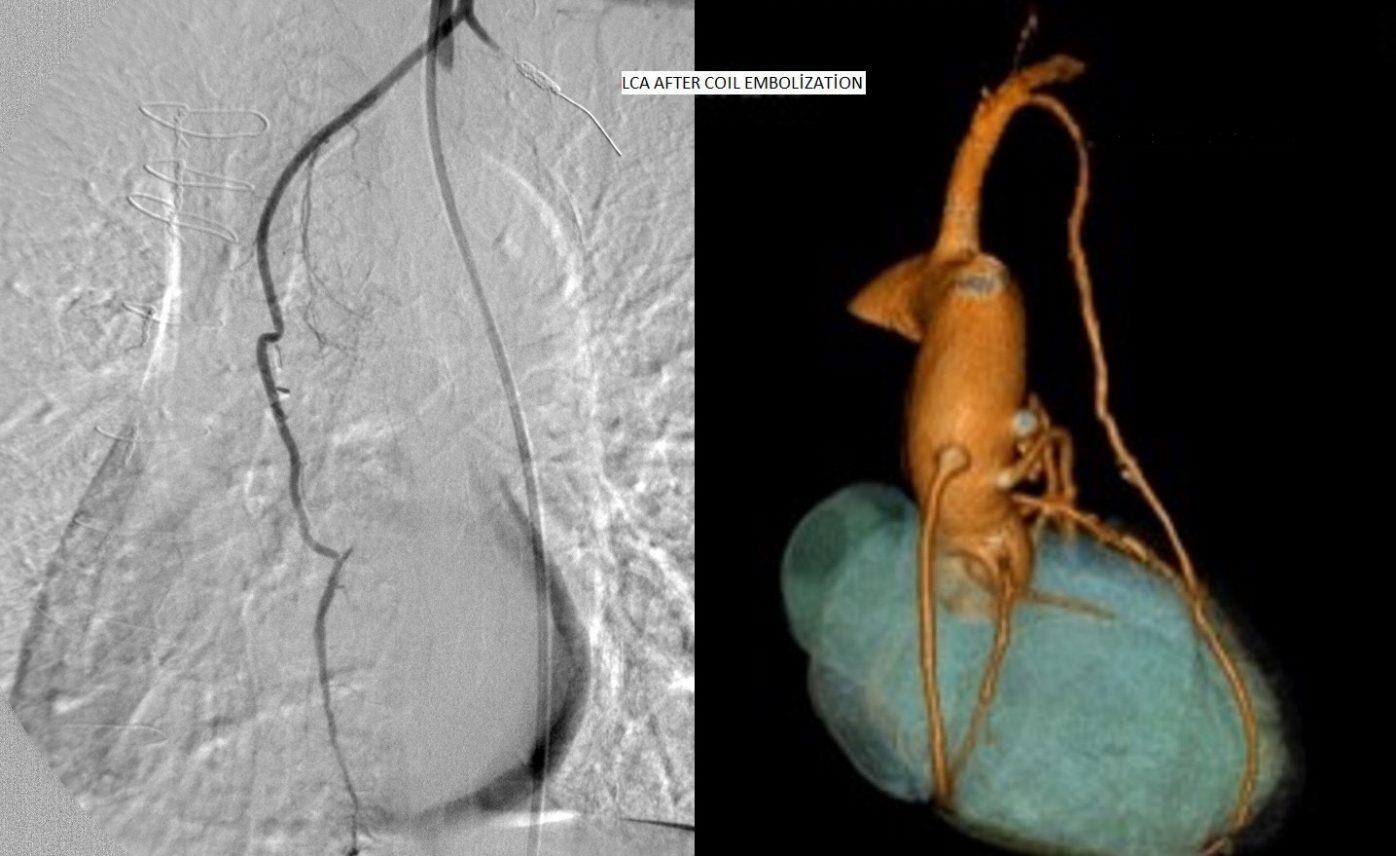
Cineangiographic and computed tomography angiographic view of the LITA after coil embolization.

Case 1: 65-year-old female patient, underwent triple CABG three months ago, applied to us with angina pectoris appearing after 50-100 m of walking. She had been under medical treatment of acetylsalicylic acid 100 mg and metoprolol 100 mg. Effort test of the patient whose physical examination and resting electrocardiography (ECG) were normal unveiled ST depression (Table 1). Coronary angiography performed in the patient revealed a well-developed LITA side branch at a distance of 2-2.5 cm from the origin of LITA (Figure 1). The accessory branch, being one and a half times the diameter of LITA, was extending to the lateral thoracic wall, where it was making anastomoses with lateral intercostal arteries and thus supplying blood to anterior and posterior side of the lateral thoracic wall. It was detected that this accessory thoracic artery, the LCA, was stealing a large part of the myocardial blood flow to lateral thoracic wall. The LCA was obliterated via coil embolization (Figure 2). The patient's effort capacity had improved and no ST segment change was observed in the effort test performed one month after the coil embolization of the lateral costal artery.

**Table 1:** Demographic characteristics of the cases.

	1.Case	2.Case	3.Case
Hemoglobin (g/dL)	12	14	11
Enzymes (U/ml)	ALT=67 Other values present no feature	LDH=440, AST=45 ALT=25, CK:450	AST=65, LDH=44 CKMB=45
Electrocardiography	ST depression	ST depression	ST elevation
Troponin T	0.04 ng/ml	0.012 ng/ml	0.45 ng/ml
Echocardiography	EF=0.40-0.45 No other feature	0.30-0.35 Enlarged right ventricle and atrium. 2^nd^ degree Mitral Insufficiency	0.45-0.50 Hypokinesia and Akinesia on the lateral wall of the left ventricle
LCA diameter (mm)	2.5	2	1.7
LITA diameter (mm)	2.1	2.3	2.5
İntraoperative LITA flow (ml/min)	45	56	48

ALT=alanine aminotransferase; LDH=lactate dehydrogenase; AST=aspartate aminotransferase; EF=ejection fraction; LCA=lateral costal artery; LITA=left internal thoracic artery

Case 2: 56-year-old female patient expressed unstable angina pectoris and dyspnea within the first week after CABG. Transthoracic ECG revealed left ventricular free wall motion abnormality and 1-2 mitral valve regurgitation. Ejection fraction was 30-35% (Table 1). Coronary angiography was performed in the patient who has been under medical treatment for diabetes mellitus for 15 years. It exposed the LCA which arose from the LITA at a distance of 2-2.5 cm from the origin of LITA. It was extending to the 6^th^ intercostal space and was two thirds the diameter of the LITA. It was postulated that the LCA had aggravated the steal phenomenon, therefore it was obliterated via coil embolization. After LCA obliteration, the patient's angina disappeared, but dyspnea persisted. Since she had advanced restrictive lung disease, she referred to a pulmonologist with medical treatment comprising of acetylsalicylic acid 100 mg, metoprolol 100 mg, spironolactone 50 mg and hydrochlorothiazide 50 mg.

Case 3: 71-year-old male patient, underwent triple CABG one month ago, applied to our emergency department with unstable angina pectoris. His ECG record displayed ST segment elevation and troponin-T value was measured 0.45 ng/ml (Table 1). In primary percutaneous coronary intervention, it was detected that the left subclavian artery (SCA) was proximally occluded, the LITA graft was patent, and there was a LITA side branch, thought to be the LCA, which was one third the diameter of the LITA. The LCA was extending to the 6^th^ rib and making anastomoses with intercostal arteries. First, balloon angioplasty was performed in the left SCA. Then, the lesion causing 80% left anterior descending artery (LAD) stenosis was stented. After that, the LCA was obliterated via coil embolization. Finally, the left SCA was stented. Stent placed in the SCA also occluded the LITA ostium inadvertently. The patient, being hemodynamically stable, was discharged from the hospital a week after admission with a medical treatment comprising of acetylsalicylic acid mg and metoprolol 100 mg. In follow-up visits, cardiac parameters have been found to be normal.

In our institution, LITA flow measurement is done by intraoperative free-bleeding technique. LITA is harvested and explored using electrocautery and metallic clips. Topical application of 0.2% papaverin solution at 37ºC is routinely done to prevent LITA spams. In the free-bleeding technique, the harvested LITA graft, before any balloon dilatation or topical papaverin application, is let to freely bleed from the distal end to a measuring cylinder for a minute while the heart rate and arterial tension are within normal limits. After measuring the total volume of blood in the cylinder, LITA graft with flow of 30 ml/min or more is considered to be proper for bypass grafting (Table 1).

## DISCUSSION

The LITA, in 92% of the cases, arises from the first part of the left subclavian artery opposite to thyrocervical trunk 2 cm above the sternal end of the clavicle. In 7% of the cases it arises from the 2^nd^ part of the left subclavian artery, whereas in 1% of the cases it does from the 3^rd^ part^[2]^. In 70% of the cases, the LITA rises directly from the left subclavian artery while in the remaining 30% it originates from the left subclavian artery as a component of a common trunk with other arteries^[2,5,6]^. After its origin from the left subclavian artery it extends on left anterior thoracic wall for 1.5 cm and 5.4 cm lateral to the sternum at the levels of the 1^st^ and 6^th^ ribs, respectively. The LITA gives pericardiophrenic, thymic, sternal, anterior intercostal and perforating branches through its course to the abdominal wall over the posterior surface of the first six ribs. It divides into musculophrenic and superior epigastric arteries at the 6^th^ intercostal space. In the cases we present, the LITA was originating from the first part of the left SCA and coursing in its natural route. Its mean diameter was 2-2.5 mm (Table 1). Perioperative manual flow measurements indicated a mean flow of 45-56 ml/min (Table 1). Tough flow and size parameters were in normal limits, the steal phenomenon seen after LAD-LITA anastomoses was ascribed to myocardial vascular resistance directing the LITA flow toward the LCA. Calafiore et al.^[7]^ in a study comparing 150 patients with left anterior thoracotomy to 150 patients with median sternotomy, reported the same rates of undivided lateral costal artery contrary to expectations, [15 (10%) and 17 (11.3%), respectively]. In the same study, rate of presence of undivided both 1^st^ intercostal artery and branches less than 1 mm in diameter were found to be significantly higher in thoracotomy group. These results indicate that the choice of incision could limit the access to smaller diameter branches but not to the LCA^[7]^. In a study comprising 262 patients who underwent CABG, Bauer et al.^[8]^ found that the LITA has large side branches in 9% of the cases and has atypical location in 1% of the cases^[8]^. The undivided LITA branches, when detected, must be obliterated since they, in direct proportion to diameter and location, reduce LITA flow. In a study comprising 38 patients with angina pectoris after CABG, Biçeroğlu et al.^[9]^ detected undivided LITA branches of varying diameter and length in 7 (18.4%) patients. Most of the side branches were found to be located at proximal parts of the LITA^[9]^. Visualization of the left SCA and the LITA before CABG is of utmost importance in the prevention of postoperative angina pectoris and myocardial infarction resulting from steal phenomenon. Otherwise, like in the cases we present, limited exploration of the LITA could result in serious complications.

A study conducted on cadavers demonstrated that the LCA shows variation at the proximal part of the LITA (15%)^[6]^. It could be present unilaterally or bilaterally, and it has a diameter close to the LITA. The same study pointed out the increased possibility of steal phenomenon due to these side branches in case the LITA was used as a vascular graft for the coronary revascularization^[6]^. Henriquez-Pino et al.^[6]^ showed that the LITA arises directly from the left SCA in 70% of the cadavers and that the internal thoracic artery gives LAC branch more distally on the left side. Other arteries accompanying the LCA at the proximal part of the LITA are the suprascapular artery, transverse cervical artery, inferior thyroidal artery, and ascending cervical artery. In the all three cases we present, the LCAs of varying diameter were anastomosing with lateral intercostal arteries. We have detected the undivided LCA in only three cases within seven years. In a long period of follow-up, due to probability of existence of asymptomatic patients and symptomatic patients applying to other institutions, the exact rate of prevalence of undivided LCA for our center couldn't be determined. In one of our cases, a female with breast-feeding history, LCA diameter was greater than the LITA diameter (Figure 1). After evaluating the coronary angiographies of 103 patients who underwent CABG surgery, Sutherland et al.^[10]^ found that the LCA was present in 30 (29%) patients, either unilaterally or bilaterally. They showed that 25 of these were extending to the 2^nd^ intercostal space, while the remaining 5 extended to the 5^th^ intercostal space.

Considering its invasive nature and potential complications, we abstained from postoperative intracoronary flow measurement. As for less invasive methods like myocardial perfusion scintigraphy, magnetic resonance imaging, positron emission tomography and transesophageal echocardiography, we faced problems regarding availability, cost, and radiation exposure. Transthoracic Doppler echocardiography is commonly used for the coronary and LITA blood flow measurements. As a result of suboptimal image quality in postoperative patient, only in the first case we were able to measure the coronary blood flow (45 cm/sn) via transthoracic Doppler echocardiography. Therefore, clinical findings and negative effort ECG were used as criteria in follow-up.

In the cases with inadequate surgical exploration of the LITA, great side branches could be passed over. LITA visualization absent in angiography could also lead to insufficient exploration of the LITA side branches. Mostly, the steal phenomenon caused by undivided LITA side branches is tried to be overcome by increasing the intensity of medical therapy, but it must be brought to mind that the presence of the LCA might be the reason for post-CABG angina.

## CONCLUSION

Considering the prevalence of LCA and undivided LCA seen after CABG, in patients planned to undergo CABG, preoperative visualization of the left SCA and proximal part of the LITA is of paramount importance. Doing this could significantly lower the probability of serious postoperative complications.

**Table:** 

**Authors’ roles & responsibilities**	
UV	Substantial contributions to the conception or design of the work; or the acquisition, analysis, or interpretation of data for the work; final approval of the version to be published
AAA	Substantial contributions to the conception or design of the work; or the acquisition, analysis, or interpretation of data for the work; final approval of the version to be published
SS	Substantial contributions to the conception or design of the work; or the acquisition, analysis, or interpretation of data for the work; final approval of the version to be published
MK	Substantial contributions to the conception or design of the work; or the acquisition, analysis, or interpretation of data for the work; final approval of the version to be published
